# Enhancing radiosensitization in EphB4 receptor-expressing Head and Neck Squamous Cell Carcinomas

**DOI:** 10.1038/srep38792

**Published:** 2016-12-12

**Authors:** Shilpa Bhatia, Kellen Hirsch, Jaspreet Sharma, Ayman Oweida, Anastacia Griego, Stephen Keysar, Antonio Jimeno, David Raben, Valery Krasnoperov, Parkash S. Gill, Elena B. Pasquale, Xiao-Jing Wang, Sana D. Karam

**Affiliations:** 1 Department of Radiation Oncology, University of Colorado Denver, Anschutz Medical Campus, Aurora, CO 80045, USA; 2 Division of Medical Oncology, School of Medicine, University of Colorado Denver, Anschutz Medical Campus, Aurora, CO 80045, USA; 3 VasGene Therapeutics Inc, Los Angeles, CA 90033, USA; 4 Division of Hematology and Pathology, University of Southern California, Los Angeles, CA 90033, USA; 5 Sanford Burnham Prebys Medical Discovery Institute, La Jolla, CA 92037, USA; 6 Department of Pathology, University of Colorado Denver, Anschutz Medical Campus, Aurora, CO 80045, USA

## Abstract

Members of the Eph family of receptor tyrosine kinases have been implicated in a wide array of human cancers. The EphB4 receptor is ubiquitously expressed in head and neck squamous cell carcinoma (HNSCC) and has been shown to impart tumorigenic and invasive characteristics to these cancers. In this study, we investigated whether EphB4 receptor targeting can enhance the radiosensitization of HNSCC. Our data show that EphB4 is expressed at high to moderate levels in HNSCC cell lines and patient-derived xenograft (PDX) tumors. We observed decreased survival fractions in HNSCC cells following EphB4 knockdown in clonogenic assays. An enhanced G2 cell cycle arrest with activation of DNA damage response pathway and increased apoptosis was evident in HNSCC cells following combined EphB4 downregulation and radiation compared to EphB4 knockdown and radiation alone. Data using HNSCC PDX models showed significant reduction in tumor volume and enhanced delay in tumor regrowth following sEphB4-HSA administration with radiation compared to single agent treatment. sEphB4-HSA is a protein known to block the interaction between the EphB4 receptor and its ephrin-B2 ligand. Overall, our findings emphasize the therapeutic relevance of EphB4 targeting as a radiosensitizer that can be exploited for the treatment of human head and neck carcinomas.

The management of locally advanced head and neck squamous cell carcinoma (HNSCC) patients presents a formidable challenge. Radiation therapy in combination with chemotherapy or targeted therapy remains the mainstay for the definitive treatment of locally advanced HNSCCs. Despite this aggressive management, there has been limited improvement in survival rates for these patients^1^,^2^. This can be attributed to activation of some of the tyrosine kinase receptor pathways that promote tumor cell proliferation and survival^3^. Initially discovered as critical players in development, emerging reports suggest that erythropoietin-producing hepatocellular carcinoma (Eph) receptors are aberrantly regulated in numerous pathological conditions including cancer^4^.

The EphB4 receptor belongs to the Eph family of receptor protein tyrosine kinases^5^ and has been shown to play a pro-tumorigenic role in carcinomas of head and neck, lung, prostate, breast, mesothelium, and esophagus^3^,^6^,^7^,^8^,^9^,^10^,^11^. Of note, EphB4 expression is limited in normal adult tissue^12^, which makes it an ideal target for therapeutic intervention. Previous studies have reported an association between EphB4 overexpression and advancement of disease^13^. Winter *et al*. have shown the presence of EphB4 on circulating tumor cells of HNSCC patients^14^. A correlation between high EphB4 expression and decreased overall survival rates in head and neck cancer patients has also been demonstrated^15^. In addition to playing a role in tumor growth, and metastasis, Eph/ephrins have also been reported to impart radioresistance to cancer cells^16^,^17^. EphB1 receptor inhibition, for example, enhances sensitivity of medulloblastoma cells to ionizing radiation both *in vitro* and *in vivo*^16^.

Based on EphB4 involvement in HNSCCs, we set to understand the role of EphB4 targeting in radiosensitization of HNSCC. We investigated whether downregulation of EphB4 expression/signaling can alter the radiosensitivity profile of HNSCCs. The underlying hypothesis is that EphB4 targeting enhances radiosensitization of HNSCC by modulating EphB4-related targets. Our findings suggest that knockdown of EphB4 modulates radiosensitivity profile *in vitro*. Similar results were observed *in vivo* using sEphB4-HSA protein with radiation. sEphB4-HSA comprises of an extracellular fragment of EphB4 receptor tagged to human serum albumin to prolong its serum half-life^18^. sEphB4-HSA acts by blocking interaction between the EphB4 receptor and the ephrin-B2 ligand^18^. The characterization, binding specificity, and pharmacokinetics of sEphB4-HSA has already been established in previous studies^18^. To our knowledge, this is the first study to elucidate the functional role of EphB4 targeting in radiosensitization of HNSCCs.

## Results

### Human HNSCC cells express high levels of EphB4 receptor

**Figure 1:**
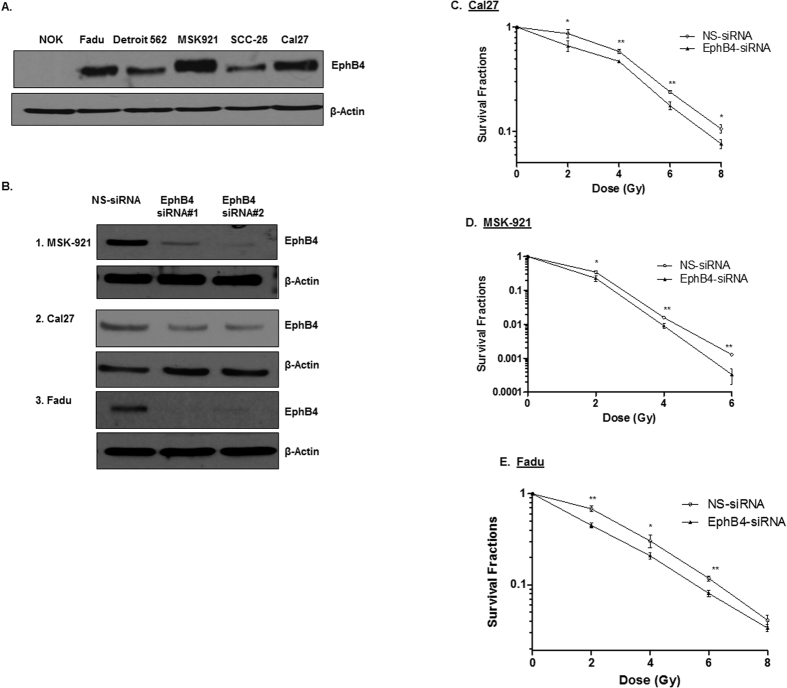
EphB4 is expressed in human HNSCC cells and its knockdown sensitizes HNSCC cells to ionizing radiation. (**A)** The EphB4 receptor is present at high to moderate levels in human HNSCC cells compared to the normal oral keratinocyte (NOK) cells as detected by Western blotting. **(B)** EphB4 expression is reduced upon transfection with the EphB4-targeting siRNAs 1 or 2 compared to the control non-specific siRNA (NS-siRNA). **(C–E)** Reduction in survival fractions in HNSCC cells is observed after transfection with the EphB4-targeting siRNA versus the control NS-siRNA (25-50 nM) in Cal27 **(C)**, MSK-921 **(D)**, and Fadu **(E)** cells as determined by clonogenic assay. Each clonogenic assay was repeated atleast three times. Representative survival plots are shown for each cell line. The survival plot for the MSK-921 cells was generated using 0-6 Gy dose of ionizing radiation because 8 Gy dose did not yield viable colonies. Data shown represent mean ± standard deviation. *p < 0.05; **p < 0.01.

The EphB4 receptor is ubiquitously expressed in HNSCCs^3^,^19^. We observed that EphB4 protein is expressed at high to moderate levels in HNSCC cells compared to normal oral keratinocyte (NOK) cells (Fig. 1A). We tested our hypothesis in the HPV negative cell lines: MSK-921, Fadu, and Cal27. Both the Fadu and the Cal27 cell lines are well characterized cell lines derived from hypopharynx and tongue respectively^20^,^21^ and display differential expression of EphB4 receptor. MSK-921 is derived from pharynx and expresses high levels of EphB4 receptor. It has been heavily explored at our institution^22^. To determine the role of EphB4 in HNSCC cells, we knocked down the expression of EphB4 using two EphB4-specific siRNAs. MSK-921, Cal-27, and Fadu cells were transfected with either EphB4-siRNAs or a control, nonspecific siRNA (NS-siRNA) and transfection efficiency was analyzed at 72 h post-transfection. We observed reduction in the EphB4 expression following knockdown by both the EphB4-targeting siRNAs compared to NS-siRNA as shown by Western blot analysis (Fig. 1B). Cells transfected with NS-siRNA did not demonstrate any obvious changes in the receptor of interest compared to non-transfected cells.

### Knockdown of EphB4 receptor enhances radiosensitization in HNSCC cells

To determine whether EphB4 knockdown can enhance the sensitivity of HNSCC cells to ionizing radiation, clonogenic survival assays were performed. We transfected HNSCC cells with an optimal dose of either control NS-siRNA or EphB4-specific siRNAs, followed by exposure to increasing doses (2, 4, 6, and 8 Gy) of radiation. After incubating cells for 9–14 days post-radiation, we analyzed the clonogenic survival fractions. Our data show that following EphB4 knockdown, HNSCC cells became more sensitive to radiation (Fig. 1C–E). In Cal27 cells, the SF2 (survival fraction at 2 Gy dose of ionizing radiation) values decreased from 0.86 in the control NS-siRNA group to 0.66 in the EphB4-siRNA group (Fig. 1C, Table 1). In MSK-921 cells, the SF2 values decreased from 0.34 in the NS-siRNA to 0.23 in the EphB4 transfected cells (Fig. 1D, Table 1). Fadu cells also demonstrated a similar trend, with SF2 values decreasing from 0.68 in the NS-siRNA transfected cells to 0.45 in the EphB4-knockdown cells (Fig. 1E, Table 1). Similar results were obtained with EphB4-siRNA#2 (data not shown).

### Combined EphB4 receptor knockdown and ionizing radiation exposure enhances G2/M cell cycle arrest

Cells display enhanced sensitivity to radiation in the G2/M phase of cell cycle^23^. Therefore, to understand the mechanism by which EphB4 knockdown might enhance the radiosensitivity of HNSCC cells, we analyzed cell cycle distribution. Flow cytometry analysis demonstrated that following combined EphB4 knockdown and radiation exposure, a higher percentage of MSK-921 cells (approx. 45%) were accumulated in the G2 phase compared to cells treated with NS-siRNA, EphB4 siRNA or NS-siRNA and radiation (8 Gy) (Supplementary Figure 1A,B). Similar trend was evident in Fadu cells, where EphB4 downregulation with radiation (6 Gy) resulted in an increased percentage of cells in the G2 phase as compared to the other experimental or control groups (Supplementary Figure 1C,D).

### Knockdown of EphB4 receptor followed by ionizing radiation induces increased DNA damage compared to single agent treatments

DNA damage is one of the main mechanisms of apoptotic cell death induced by radiation^24^. To investigate whether EphB4 knockdown radiosensitizes HNSCC cells by affecting DNA damage response pathways, we studied cellular induction and accumulation of γ-H2AX, a hallmark of the DNA damage response^24^, by both immunofluorescence and flow cytometry approaches in different HNSCC cell lines. The results from immunofluorescence were in concordance with flow cytometry.

**Figure 2:**
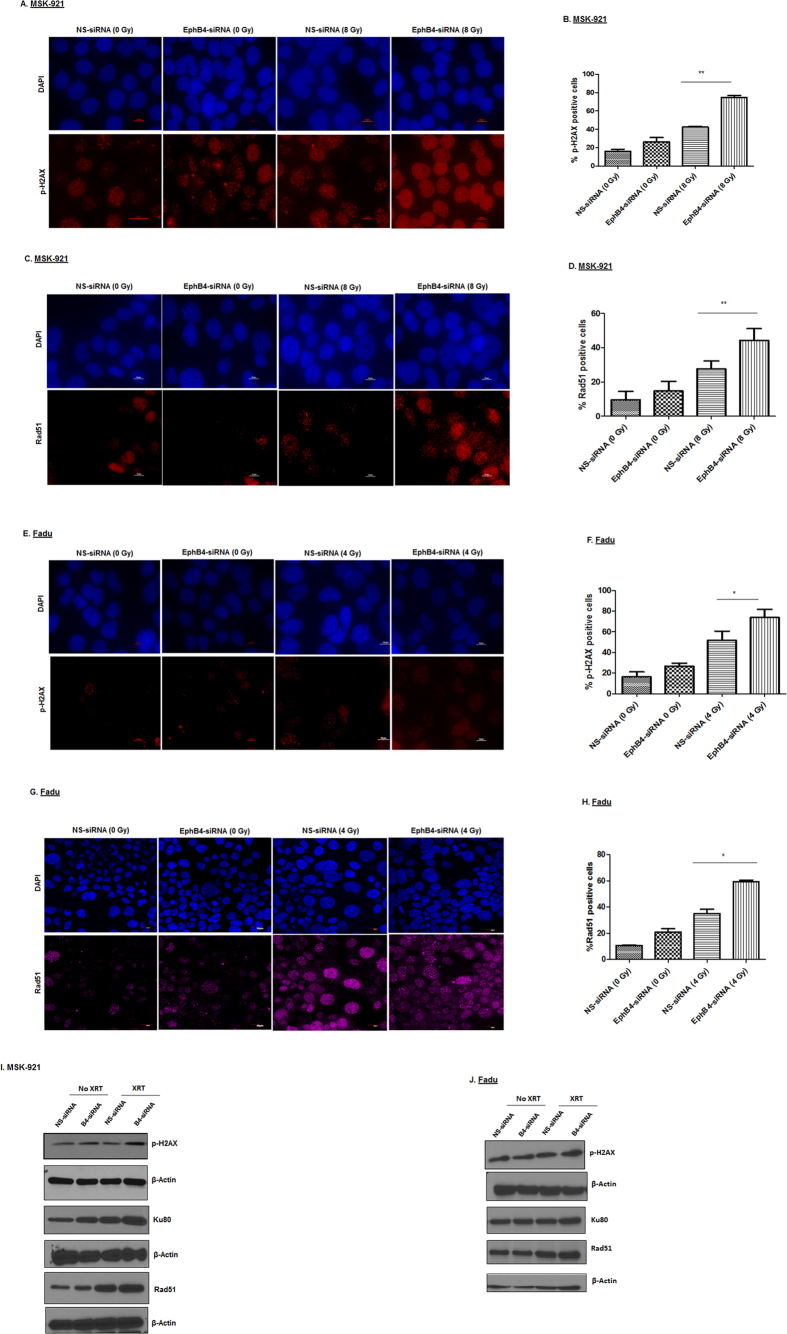
EphB4 downregulation combined with ionizing radiation enhances the DNA damage response in HNSCC cells. p-H2AX analysis shows a significant enhancement in the percentage of p-H2AX positive MSK-921 cells following EphB4 knockdown and ionizing radiation as demonstrated by immunofluorescence staining **(A** and **B)**. In addition, an increase in Rad51-foci is also evident in MSK-921 cells **(C** and **D).** A similar trend was observed in Fadu cells in terms of p-H2AX **(E** and **F)** and Rad51 **(G** and **H)** expression. Data shown represent mean ± standard error from two to three independent experiments. *p < 0.05; **p < 0.005. Total magnification: 600–1000x. Western blot analyses demonstrate enhanced levels of p-H2AX, Ku80, and Rad51 indicative of the DNA damage response in the combination group compared to the other experimental groups in MSK-921 cells **(I)** and Fadu cells **(J)**.

By immunofluorescence staining with anti-p-H2AX antibody, we observed ~20–30% increase in γ-H2AX foci with the addition of EphB4 knockdown to radiation treatment in MSK-921 cells (Fig. 2A,B, p < 0.005). We also analyzed another DNA damage response protein, Rad51 and found that combined EphB4 knockdown and radiation resulted in ~20% enhancement in Rad51 foci in MSK-921 cell line (Fig. 2C,D, p < 0.005) compared to radiation alone. Similar results were evident in Fadu cells in combination group compared to radiation alone treatment with respect to p-H2AX expression (Fig. 2E,F, p < 0.05) and Rad51 (Fig. 2G,H, p < 0.05). These data for p-H2AX expression were corroborated by flow cytometry where EphB4 knockdown in combination with radiation induced an ~10% increase in p-H2AX-expressing cells compared to radiation in both MSK-921 (Supplementary Figure 2A, p < 0.05) and Cal27 cells (Supplementary Figure 2B).

To further explore key players of the DNA damage response pathway, we performed Western blot analysis. We observed increased expression of p-H2AX, Rad51, and Ku80 following EphB4 knockdown in MSK-921 cells (Fig. 2I) and Fadu cells (Fig. 2J) that were irradiated with 8 Gy dose compared to other groups at 24 h post- radiation.

### Combined EphB4 knockdown and ionizing radiation induces enhanced apoptosis in HNSCC cells

**Figure 3:**
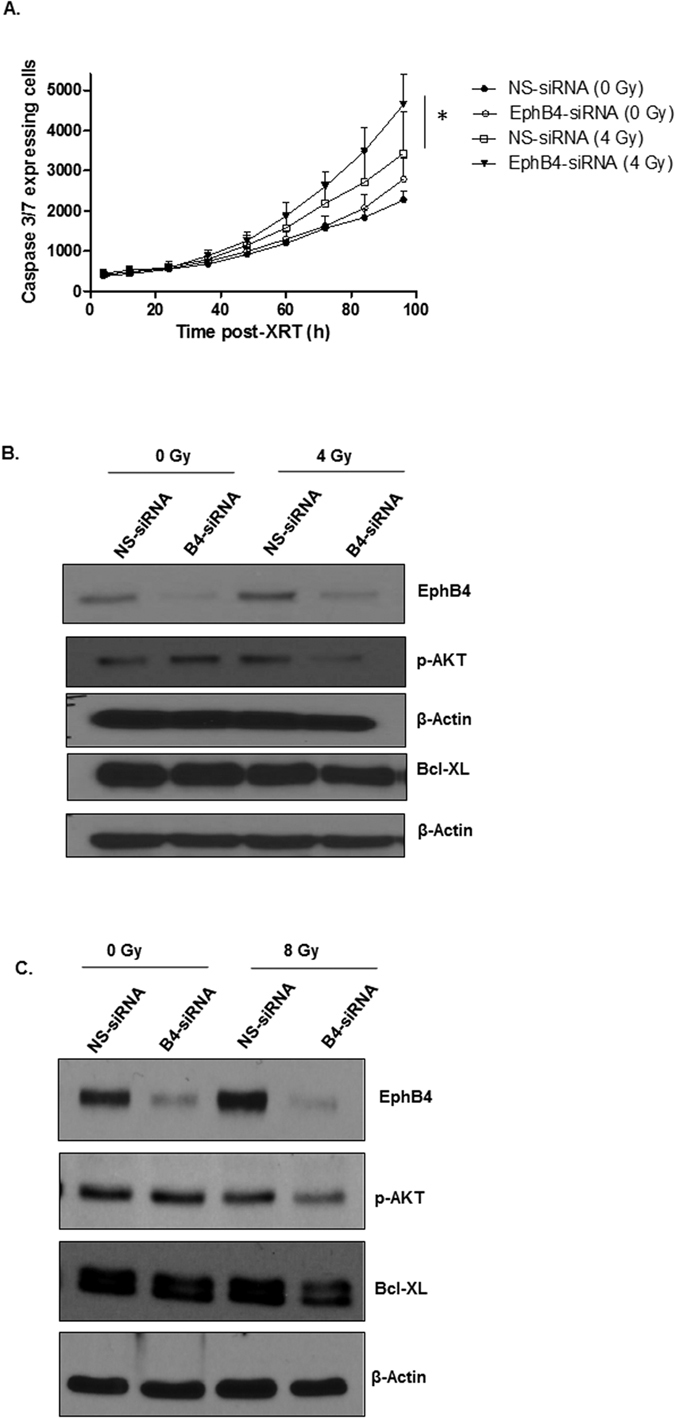
Combined EphB4 knockdown and ionizing radiation exposure induces apoptosis in HNSCC cells. EphB4 knockdown combined with radiation enhances apoptosis in Fadu cells as shown by caspase 3/7 assay **(A)**. Western blot analysis show modulation in the levels of pro-survival markers in Fadu **(B)** and MSK-921 **(C)** cells following EphB4 knockdown and radiation exposure (XRT). Each experiment was repeated atleast two times. Data shown represent mean ± standard error from two independent experiments. *p < 0.05.

Enhanced apoptosis is one of the key anti-tumor responses mediated by EphB4 targeting and radiation^3^,^25^,^26^,^27^,^28^. In caspase-3/7 assay, we observed a significant increase in apoptosis (~28%) in Fadu cells transfected with EphB4-siRNA and 4 Gy dose of radiation compared to radiation alone at 96 h (Fig. 3A, p < 0.05). When we used this assay in MSK-921 cells, combining EphB4-siRNA treatment with radiation enhanced apoptosis compared to EphB4-siRNA alone only (data not shown). We next analyzed the expression of pro-survival markers such as p-AKT and Bcl-XL in both Fadu and MSK-921 cells following EphB4-siRNA or control NS-siRNA transfection +/− radiation. Our western blot data show decreased levels of both p-AKT, and Bcl-XL proteins in the combined treatment group compared to other groups at 72 h post-radiation in Fadu (Fig. 3B) and MSK-921 cells (Fig. 3C).

Eph receptors are reported to interact with other tyrosine kinase receptors such as EGFR to promote cell survival and metastasis^29^. We observed that phosphorylated EGFR (p-EGFR) and total EGFR levels were reduced in the combined EphB4 knockdown and radiation group compared to other groups in MSK-921 cells (Supplementary Figure 3).

### sEphB4-HSA treatment enhances radiosensitization in PDX models of HNSCC

To examine the impact of EphB4 targeting on radiosensitivity *in vivo*, we used three PDX models of HNSCC. Some of the characteristics of these tumors are listed in Supplementary Table 1. These patient-derived tumors were found to express high levels of EphB4 (Supplementary Figure 4). Small pieces of tumor tissue were implanted in the flanks of nude mice as described in the materials and methods. Once the tumors reached 50–150 mm^3^, mice were randomized into four groups (n = 6–8 per group), including (1) PBS, (2) sEphB4-HSA, (3) PBS + radiation (XRT) and (4) sEphB4-HSA + radiation (XRT). We utilized a 5 Gy x 4 fractions radiation dosing delivered bi-weekly over a period of two weeks. To rule out a false negative radiosensitization effect due to high radiation dose that might have eliminated an effect in CUHN022 tumors, we also employed dose de-intensification to 2 Gy x 5 fractions.

**Figure 4:**
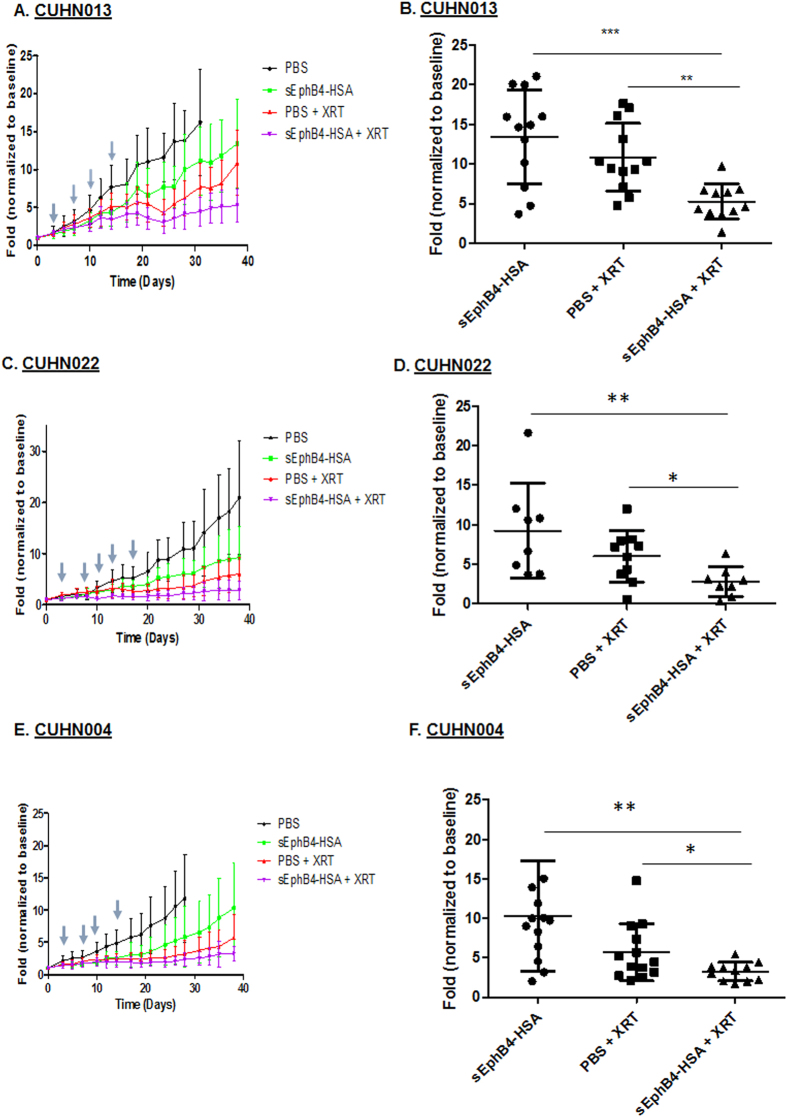
EphB4 targeting enhances radiosensitivity in HNSCC PDX models. Tumor growth analyses of CUHN013 PDX tumors **(A,B)**, CUHN022 PDX tumors **(C,D)**, and CUHN004 PDX tumors **(E,F)** show that sEphB4-HSA treatment combined with radiation significantly decreases tumor volume over time compared to single agent treatments. sEphB4-HSA was administered on days 0, 3, 5, 7, 10, 12, 14, 17, 19, 21, 24, 26, 28, 31, 33, 35, and 38. The symbol “↓” represent days when tumors were exposed to ionizing radiation (5 Gy/fraction for CUHN013 and CUHN004; 2 Gy/fraction for CUHN022). The mice in the PBS group were sacrificed on day 31 (CUHN013), or day 28 (CUHN004) because their tumors became too large. Data shown represent mean ± standard deviation. *p < 0.05,**p < 0.005, **p < 0.0005.

We observed that sEphB4-HSA treatment decreased tumor growth in mice implanted with CUHN013, CUHN022, and CUHN004 tumors (Fig. 4A,C and E). Fractionated ionizing radiation (PBS+XRT) significantly reduced tumor volumes in mice engrafted with CUHN013, and CUHN004 tumors. Furthermore, CUHN013 tumor growth analysis showed a significant reduction in tumor volume by approximately 4 fold following sEphB4-HSA treatment + XRT compared to PBS+XRT on day 38 post-treatment (Fig. 4B, p < 0.005). For CUHN004 tumors, combining radiation with sEphB4-HSA showed significant tumor growth reduction by approximately 3 fold compared to PBS+XRT on day 38 (Fig. 4E,F; p < 0.05). In mice implanted with the HPV+ tumor, CUHN022, there was no significant impact of the combination treatment compared to radiation alone at 5 Gy dose (Supplementary Figure 5 A,B). Given that HPV positive tumors are known to be exquisitely radiosensitive, we reduced the radiation dose to examine if this would yield a radiosensitization effect. Figure 4C and D show that tumor growth was reduced by approximately 0.5 fold in the combination group compared to PBS+XRT on day 34 post-XRT.

Since sEphB4-HSA acts by blocking the binding of EphB4 with its ligand ephrin-B2, we performed ELISA measuring EphB4 tyrosine phosphorylation (activation) in PDX tumors to demonstrate successful EphB4 targeting *in vivo*. Our data showed a significant decrease in p-EphB4 in CUHN004 tumors treated with sEphB4-HSA compared to control PBS group (Supplementary Figure 6).

### EphB4 targeting radiosensitizes HNSCC PDX tumors by affecting proliferation, and apoptotic pathways

**Figure 5:**
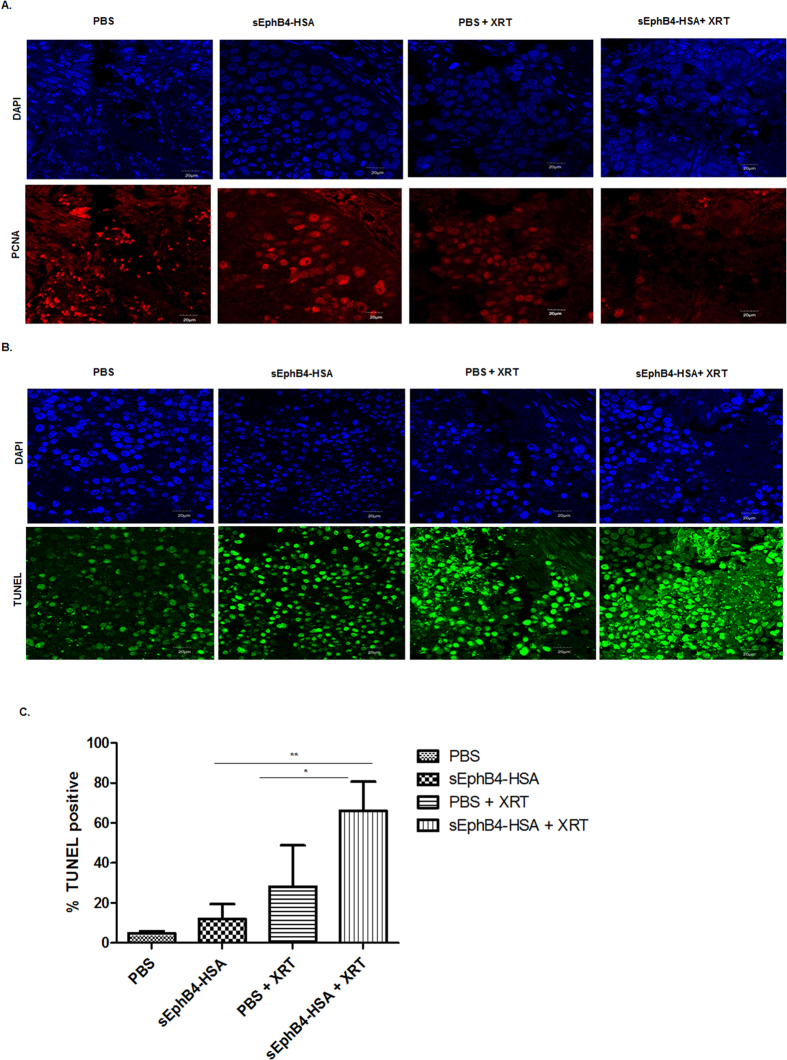
EphB4 targeting radiosensitizes HNSCC tumors and affects tumor growth by reducing proliferation, and enhancing apoptosis. Immunofluorescence analysis of CUHN013 PDX tumors show reduction in PCNA (**A**) and enhancement in TUNEL-positive nuclei (**B**) in mice treated with sEphB4-HSA and ionizing radiation (XRT) compared to mice treated with a single agent. Quantitative analysis of TUNEL staining (**C**) show significant increase in apoptosis as evident by TUNEL-positive nuclei in the combination group compared to single agents. Data shown represent mean ± standard deviation. *p < 0.05, **p < 0.005. Total magnification: 600x.

**Figure 6:**
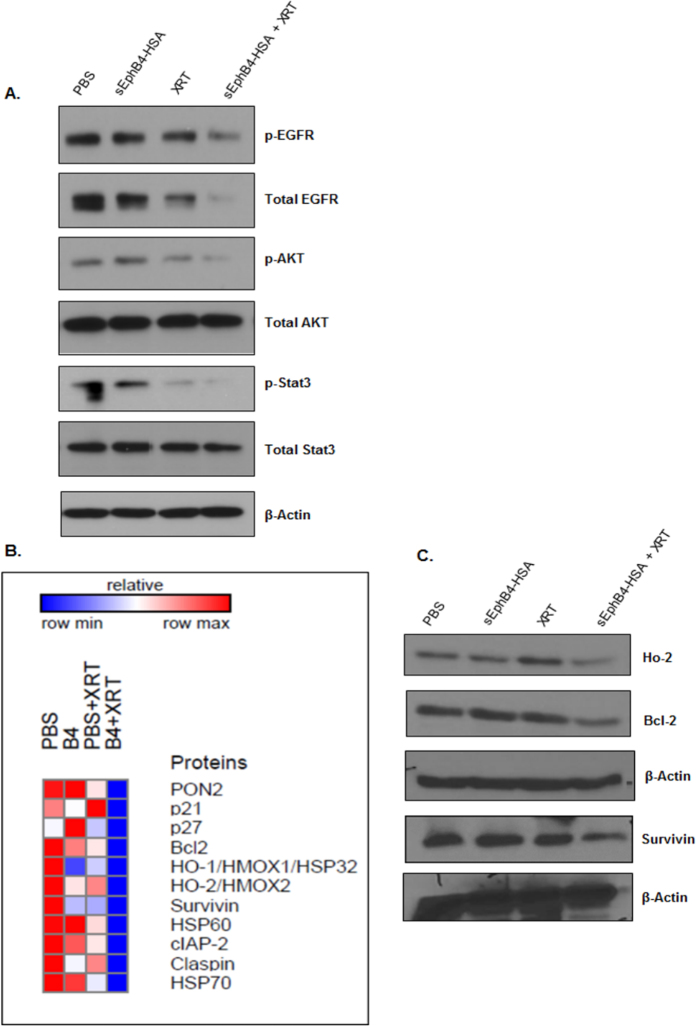
EphB4 targeting in combination with ionizing radiation exposure alters the expression of apoptotic and tumor growth promoting molecules in CUHN013 tumors. Western blot analysis shows decreased phosphorylation or decreased levels of proteins implicated in tumor growth and cell survival in CUHN013 tumors treated with sEphB4-HSA and radiation compared to tumors treated with a single agent. (**B**) A heat map generated based on the results of a human apoptosis antibody array using GeneE software (Broad institute, USA) depicts decreased expression of survival markers in CUHN013 tumors treated as indicated. (**C**) Validation of hits by western blotting shows decreased levels of pro-survival proteins such as Ho-2, Bcl-2, and survivin in tumors treated with sEphB4-HSA combined with radiation (XRT) compared to tumors treated with a single agent.

Immunofluorescence analysis in PDX tumors showed reduced expression of the proliferation marker PCNA in the combination group compared to other groups (Fig. 5A). To further explore the mechanism of radiosensitization *in vivo*, we performed TUNEL assay. A significant enhancement in percentage of the TUNEL-positive nuclei was observed following combined sEphB4-HSA and radiation treatment compared to single treatments in the CUHN013 tumors (Fig. 5B,C). Western blot analysis show decreased expression of p-EGFR, EGFR, p-STAT3, STAT-3, and p-AKT in tumors treated with sEphB4-HSA and radiation compared to single agent or control PBS (Fig. 6A). We also used a human apoptosis antibody array to delineate apoptotic proteins that are modulated as a result of EphB4 targeting +/−XRT (Fig. 6B). Our data show a decrease in the levels of Bcl-2, survivin, and Ho-2 proteins in the combination group (sEphB4-HSA + XRT) compared to other groups in CUHN013 tumors (Fig. 6C).

## Discussion

Eph/ephrin signaling is dysregulated in a number of human cancers including head and neck squamous cell carcinomas^30^. Accumulating evidence suggests that Eph/ephrin family members including EphB1, EphA2, EphB4, ephrin-A1, and ephrin-A3 impart radioresistant phenotype to the cancer cells^16^,^17^,^31^,^32^,^33^. In the present study, we investigated the functional significance of EphB4 targeting/knockdown in enhancing radiosensitivity of HNSCCs. Our data indicate that EphB4 knockdown enhances cellular radiosensitization by decreasing clonogenic survival in HNSCC cells, inducing G2 cell cycle arrest, modulating the DNA damage response pathway, and ultimately resulting in apoptotic cell death. The HNSCC cells showed reduction in clonogenic survival following transfection with EphB4-siRNA vs. control NS-siRNA at increasing radiation doses. The differences in the clonogenic profile can be partly attributed to the differential expression of the EphB4 receptor present on HNSCC cells. Furthermore, enhanced radiosensitization effect observed after EphB4 downregulation is mediated in part *via* increased G2 cell cycle arrest, which was found to be radiation dose-dependent.

The *in vitro* findings were substantiated *in vivo* by HNSCC PDX models. The combination of radiation and EphB4 targeting used not only resulted in a significant reduction in tumor volume but also delayed tumor growth compared to single agent treatments. This was evident in tumors derived from HPV-negative patients with aggressive histology, heavy smoking history, who had failed chemoionizing radiation therapy (CUHN013, and CUHN004) and HPV-positive never-smoker patient (CUHN022). In fact, we observed an interesting synergistic response when we combined EphB4 targeting with radiation together. In CUHN022 tumor, radiosensitization due to EphB4 targeting was observed after radiation dose de-escalation. HPV-positive tumors are known to be less malignant^2^,^34^ and ongoing trials are focused on treatment de-intensification^35^,^36^,^37^,^38^. Our results with CUHN022 show no effect of EphB4 targeting on radiosensitization with high dose of radiation, but a difference was noticed when the radiation dose was decreased. This could be important in achieving maximal therapeutic effect while minimizing radiation-induced toxicity, particularly at high doses. Furthermore, our mechanistic data supports the hypothesis that combined sEphB4-HSA and radiation treatment results in enhanced radiosensitization of HNSCC tumors. The radiosensitization effect is mediated *via* effects on cell proliferation and cell survival pathways. Thus, our results are in agreement with published reports suggesting a role of EphB4 in HNSCC^3^.

Enhanced apoptosis is one of the main mechanisms underlying the anti-tumorigenic effects of sEphB4-HSA treatment^3^,^25^,^26^,^39^,^40^. Reports suggest that DNA damage is a causative factor of apoptosis following radiation^24^. Our immunofluorescence and flow cytometry data are the first to show that EphB4 knockdown along with radiation results in significant enhancement in the levels of p-H2AX, a DNA damage sensor protein. We also noticed an increase in the number of Rad51-positive cells by immunofluorescence staining following combined EphB4 downregulation and radiation compared to either treatment alone. We observed an increased expression of DNA damage response proteins (including p-H2AX, Ku80, and Rad51 protein expression) in HNSCC cells following EphB4 knockdown in the absence or presence of radiation. This is in agreement with a study that suggests the involvement of Eph receptors and ephrins in the DNA damage response^41^. A correlation was shown between Eph/ephrin expression in human cancers with increased DNA damage repair and blockade of apoptosis^41^. Another study documented the role of Dasatinib, a tyrosine kinase inhibitor that targets EphB4, EphA2, and EphB2 among other kinases^42^,^43^, in exerting a radiosensitizing effect by affecting the DNA double-strand break response pathway. Our findings indicate that the radiosensitization effect observed following EphB4 knockdown is partly mediated *via* modulation of DNA damage response pathway.

Previous studies have found a correlation between radiation and programmed cell death^27^,^28^,^44^,^45^. Importantly, EphB4 knockdown has been reported to promote cell death by apoptosis^3^,^39^. We used a caspase3/7 assay to measure apoptotic cell death in HNSCC cells. In addition, we used an apoptosis antibody array to analyze PDX tumors and identify apoptotic markers that could be modulated following sEphB4-HSA and radiation treatment. Our data suggest a significant increase in caspase3/7 expressing cells after combined EphB4 knockdown and radiation treatment compared to other groups. This was accompanied by decrease in the levels of pro-survival markers such as Bcl-XL, and p-AKT in HNSCC cells. Our findings suggest that the DNA damage response pathway stimulated in response to EphB4 knockdown and radiation ultimately results in tumor cell death. Consistent with our *in vitro* results, we noticed a significant increase in TUNEL staining, which was accompanied by decreased levels of anti-apoptotic proteins such as Bcl-2, Ho-2, and survivin in the tumors harvested from combination treatment group compared to single agent alone. We also observed a decreased expression of the proliferation marker, PCNA, in the combined treatment group compared to groups treated with single agents *in vivo*.

The role of EGFR signaling in tumorigenesis is well documented^46^. Our results show decreased levels of both phosphorylated EGFR, and total EGFR following EphB4-siRNA transfection and radiation exposure in MSK-921 cells, suggesting that functional interaction between the EphB4 receptor and EGFR might be responsible for promoting tumorigenesis and progression in HNSCC cells. Tumors harvested from the combination group (sEphB4-HSA + XRT) had lower levels of both p-EGFR and total EGFR compared to other groups. Since several human cancers have elevated expression of EGFR along with Eph receptors, therapeutic agents targeted against Eph family members could potentially also affect tumors through EGFR inhibition. We are currently expanding on this finding in an ongoing project in our laboratory. In addition, we observed reduced expression of another pro-survival protein such as p-AKT in tumors exposed to combination treatment compared to either treatment alone. We also observed a significant decrease in the levels of phosphorylated EphB4 in PDX tumors in the sEphB4-HSA administered group compared to the control PBS group. Targeting of EphB4 axis may not be the only mechanism of sEphB4-HSA protein. Abrogation of other Eph receptors and of ephrin-B2 reverse signaling may also play a role. Studies are currently underway to determine how disrupting the interaction between Eph receptor and its cognate ligand affects tumor growth in HNSCCs. The signal transducer and activator of transcription-3 (STAT3) pathway plays a critical role in stimulating proliferation, invasion, and evasion of apoptosis in human cancers including cancers of head and neck^47^,^48^. Ferguson *et al*. have shown that in lung carcinoma cells, EphB4 knockdown affects apoptosis by altering the expression of the JAK-STAT family of proteins^25^. Another study by Pradeep *et al*. reported that EphB4 receptor promotes tumor growth and progression *via* stimulation of STAT3 signaling^26^. We noticed reduced expression of phosphorylated and total STAT3 in PDX tumors following sEphB4-HSA treatment with radiation. This data suggest that EphB4 targeting combined with radiation acts by affecting EphB4 downstream targets, ultimately resulting in an anti-tumor response observed in the form of tumor growth delay and significant reduction in tumor volumes in HNSCC PDX tumors.

Overall, our findings underscore the importance of EphB4 targeting in enhancing radiosensitization in both HNSCC cells and PDX models. The effect is mediated by alterations in cell cycle, DNA damage, and cell death pathways. Studies are currently underway to screen a broad range of PDX tumors and categorize them into different subtypes. From a translational viewpoint, the information obtained from these studies would be very useful for predicting the treatment responses of HNSCC patients and eventually might have an impact on personalized patient care. In conclusion, our data suggest that EphB4 serves as an ideal target and EphB4-directed therapeutic agents in combination with radiation may hold a great promise for clinical translation in head and neck cancer.

## Materials and Methods

### Cell lines and reagents

The human HNSCC cell lines Cal27, and Fadu were obtained from the American Type Culture Collection (ATCC, Rockville, MD, USA). MSK-921, SCC-25, Detroit 562, and normal oral keratinocyte (NOK) cell lines were obtained from Dr. XJ Wang’s lab (University of Colorado, Anschutz Medical Campus, Aurora, CO, USA). MSK-921 cells were maintained in RPMI-1640 medium with 10% fetal bovine serum, and primocin (Invivogen, San Diego, CA, USA). Cal27, Fadu, SCC-25, and Detroit 562 cells were maintained in Dulbecco’s Modified Eagle’s Medium (DMEM) with 10% fetal bovine serum, primocin at 37 °C and 5% CO_2_. NOK cells were grown in defined keratinocyte medium (Gibco, NY, USA). sEphB4-HSA protein was provided by Vasgene Therapeutics Inc. (Los Angeles, CA, USA).

### siRNA Transfection

For transfection, two EphB4 targeting siRNAs and a control non-specific siRNA were used. HNSCC cells were transfected in serum-free, antibiotic-free growth medium using Mirus TransIT-TKO Transfection Reagent (Madison, WI, USA), according to the manufacturer’s instructions. Short interfering RNAs (siRNA) specific for human *EphB4* (ID: s243, and 533) and the non-specific control siRNA were obtained from Invitrogen (Carlsbad, CA, USA). Briefly, cells were transfected using 10 μL TransIT-TKO for a final concentration of 25–50 nM siRNA. Cells were incubated with the transfection complex for 4–20 h, medium was replaced with fresh serum-containing and antibiotic-containing growth medium and used for further analysis.

### Whole cell lysate preparation

Human HNSCC cells were homogenized in RIPA buffer (Millipore, MA, USA), containing protease inhibitor cocktail (Thermo Fisher Scientific Inc., IL, USA) and phosphatase inhibitors (Sigma, MO, USA) on ice for 30 min. Lysates were collected and protein concentration was determined as described^16^.

### Western blotting and antibodies

Protein lysates (20–30 μg) were loaded onto 10–12% SDS-PAGE gels, electrophoresis, and blocking were conducted as described^16^. Blots were probed overnight at 4 °C with respective antibodies. Primary antibodies (anti-p-Akt, anti-p-EGFR, anti-Bcl-XL, anti-Ku80, anti-p-STAT3, STAT3, anti-p-H2AX, and anti-β-actin) were obtained from Cell Signaling Technology (Danvers, MA, USA). Anti-EGFR and anti-Rad51 antibody was purchased from Santa Cruz Biotechnology, Dallas, TX, USA, and anti-EphB4 antibody (clone m265) was provided by Vasgene Therapeutics Inc. (Los Angeles, CA, USA). Horseradish peroxidase (HRP)–conjugated secondary antibodies were obtained from Sigma (St. Louis, MO, USA).

### Irradiation

Cells and animals were irradiated with indicated radiation doses using a 160 KVp source RS-2000 (Rad Source Technologies, Inc) X-ray irradiator at 25 mAmp, and at a dose rate of 1.24 Gy/minute. The biological irradiator used for both *in vitro* and *in vivo* experiments has a 0.3 mm copper filter. A customized shield exposing only the flank tumors was used to irradiate mice.

### Clonogenic survival assay

Cellular survival was determined following exposure of cells to ionizing radiation in 25 cm^3^ flasks. Clonogenic cell survival was analyzed as described^49^,^50^. Briefly, colonies of >50 cells were counted 9–14 days after radiation. Plating efficiency (PE) and survival fraction (SF) were calculated using the following formulas:

PE = Number of colonies formed/Number of cells seeded

SF = Number of colonies formed after radiation/Number of cells seeded × PE.

Survival fraction following radiation in NS-siRNA or EphB4-siRNA transfected cells was normalized taking into account plating efficiency in that particular group at 0 Gy. Each experiment was replicated atleast 3 times.

### Cell cycle analysis

MSK-921 and Fadu cells were seeded at a density of 150,000 cells/well in 6-well plates. Cells were transfected using the transfection protocol described above and irradiated with an optimal radiation dose. After 48–72 h radiation, cells were collected, washed twice in ice-cold PBS, fixed in 70% ethanol, stained with propidium iodide, and analyzed for cell cycle by flow cytometer. Each experiment was repeated 2–3 times.

### DNA damage analysis

#### Immunofluorescence staining

HNSCC cells were plated in 8-well chamber slides (30,000 cells/well) in 10% growth medium containing primocin. Cells were transfected using the protocol described earlier. At 48 hours post-plating, cells were left un-radiated or irradiated with an optimal dose of radiation and were analyzed 4 h later following incubation with anti-p-H2AX antibody (1:2000 dilution) or analyzed at 12–24 h after incubation with anti-Rad51 antibody (1:50 dilution, Santa Cruz Biotechnology, Dallas, TX, USA). This step was followed by incubation with AlexaFlour-560 or AlexaFlour-647 IgG secondary antibody (1:500 dilution, Life Technologies, Carlsbald, CA, USA). Images were captured using a 60x or 100x oil objective on a Nikon fluorescence or Olympus confocal microscope. Cells with more than five foci were counted as positive. The relative percentage of positive cells was determined in 10 fields using the formula: (Number of positive cells per field/Total number of cells)*100. Each experiment was replicated atleast 2–3 times.

#### Flow cytometry analysis

MSK-921 cells (200,000 cells/well) and Cal27 cells (150,000 cells/well) were seeded in 6-well plates. Cells were transfected using the protocol described above. After 24 h, cells were left unexposed or exposed to either 8 Gy dose (MSK-921 cells) or 4 Gy dose (Cal27 cells) of radiation. After 4 h, cells were collected by trypsinization. Samples were washed with PBS and labeled with Fixable Viability Dye eFluor 506 (eBioscience, San Diego, CA, USA) for 30 minutes on ice. Samples were then washed, fixed using the Foxp3 Staining Buffer Set (eBioscience, San Diego, CA, USA) followed by staining with Anti-Hu/Mo pH2AX eFluor 660 (eBioscience, San Diego, CA, USA) at room temperature for 30 minutes. Samples were washed with PBS and analyzed by flow cytometry. Each experiment was replicated 2–3 times.

### Caspase 3/7 activity assay

Fadu cells were seeded at a density of 200,000 cells/well in a 6-well plate and transfected using the protocol described above. At approximately 24 h after transfection, medium was replaced and cells were replated at a density of 1000 cells/well in a 96-well plate. After 24 h, cells were either left non-radiated or irradiated using a 8 Gy dose of radiation and caspase 3/7 reagent (Essen Bioscience, Ann Arbor, MI, USA) was added to the wells at 1:1000 dilution. Caspase 3/7 activity was monitored in real-time using an incucyte machine and plotted as mean values from multiple replicates per experimental condition. The experiment was replicated 2 times.

### *In vivo* PDX studies and radiosensitization experiments

Female athymic nude mice (5–6 weeks old) were purchased from Envigo (Indianapolis, In, USA). All mice were handled and euthanized in accordance with the ethics guidelines and conditions set and overseen by the University of Colorado, Anschutz Medical Campus Animal Care and Use Committee. All protocols for animal studies were reviewed and approved by the Institutional Animal Care and Use committee at the University of Colorado, Anschutz Medical Campus. HNSCC PDX tumors (F8-F16 generation) were obtained from Dr. Antonio Jimeno’s lab (University of Colorado, Anschutz Medical Campus). For implantation, tumors were cut into approximately 3 × 3 × 3 mm pieces. Upto 30 mice (60 tumors) were implanted in each experiment. The right and left hind flanks were sterilized and small incisions were made to create subcutaneous pocket. Tumor pieces were dipped in Matrigel (BD Biosciences, San Jose, CA, USA) and inserted into the subcutaneous pocket. Tumor growth was measured using a digital caliper and tumor volume was calculated using the formula: [(smaller diameter)^2^ × (longer diameter)]/2. When tumor volumes reached approximately 50–150 mm^3^, mice were randomized into four groups (1) PBS, (2) sEphB4-HSA, (3) PBS + XRT, and (4) sEphB4-HSA + XRT. Mice were then either injected intraperitoneally with PBS or with a 20 mg/kg dose of sEphB4-HSA three times/week throughout the experiment. Following first injection of sEphB4-HSA, mice were treated with radiation (5 Gy/fraction x 4 fractions or 2 Gy/fraction x 5 fractions) using an X-ray irradiator. The mice were irradiated two times/week for a period of two weeks. Fold differences in tumor volume were calculated by normalizing individual tumor volumes to the volume measured at day 0. The statistical significance on tumor growth curves between PBS+XRT and sEphB4-HSA + XRT groups was assessed by Student’s t-test using the GraphPad Prism 4.0 software. A p-value of < 0.05 was considered statistically significant. At the end of the experiment, tumors were collected, flash-frozen or formalin-fixed for immunofluorescence and Western blot analysis.

### Immunofluorescence staining

Immunofluorescence staining was performed on tumors harvested from different groups implanted with CUHN013 tumors using anti-PCNA antibody (1:200 dilution, BD biosciences, San Jose, CA, USA). This was followed by incubation with AlexaFlour-560 IgG secondary antibody (1:500 dilution, Life Technologies, Carlsbald, CA, USA). Images were captured using a 60x objective on Olympus confocal microscope.

### ELISA assay

Protein lysates were collected from CUHN004 tumors subjected to different treatment conditions as described earlier^16^. p-EphB4 levels in the tumor samples were measured using Human p-EphB4 ELISA kit (R&D systems, Minneapolis, MN, USA) following manufacturer’s instructions.

### Human apoptosis antibody array

Human apoptosis antibody array kit was purchased from R&D Systems (Minneapolis, MN, USA). Tumors were homogenized as described above. Lysates were prepared and incubated with the array as per manufacturer’s instructions. Following addition of chemiluminescent detection reagents, a signal proportional to the amount of protein bound was detected.

### TUNEL assay

We performed TUNEL assay on PDX tumors using *in situ* cell death detection kit (Roche, Indianapolis, IN, USA). Images were captured using Olympus confocal microscope. Atleast 4–5 fields were chosen for quantitative analysis by Image J software.

### Statistical analysis

All the experiments were performed in duplicate or triplicate and repeated 2–3 times. Quantitative analyses were performed using Student’s t-test or ANOVA. A p-value of < 0.05 was considered significant.

## Additional Information

**How to cite this article**: Bhatia, S. *et al*. Enhancing radiosensitization in EphB4 receptor-expressing Head and Neck Squamous Cell Carcinomas. *Sci. Rep.* **6**, 38792; doi: 10.1038/srep38792 (2016).

**Publisher's note:** Springer Nature remains neutral with regard to jurisdictional claims in published maps and institutional affiliations.

## Supplementary Material

Supplementary Data

## Figures and Tables

**Table 1:** This table lists the survival fractions at 2 Gy dose of ionizing radiation (SF2) for each individual cell line comparing effects of the EphB4-siRNA transfection to those of the NS-siRNA transfection.

Cell lines	NS-siRNA (SF2 values)	EphB4-siRNA (SF2 values)
Cal27	0.86	0.66
MSK-921	0.34	0.23
Fadu	0.68	0.45

## References

[b1] Ang K. K. . Randomized phase III trial of concurrent accelerated radiation plus cisplatin with or without cetuximab for stage III to IV head and neck carcinoma: RTOG 0522. Journal of clinical oncology: official journal of the American Society of Clinical Oncology 32, 2940–2950, doi: 10.1200/JCO.2013.53.5633 (2014). 25154822 PMC4162493

[b2] Nguyen-Tan P. F. . Randomized phase III trial to test accelerated versus standard fractionation in combination with concurrent cisplatin for head and neck carcinomas in the Radiation Therapy Oncology Group 0129 trial: long-term report of efficacy and toxicity. J Clin Oncol 32, 3858–3866, doi: 10.1200/JCO.2014.55.3925 (2014). 25366680 PMC4239304

[b3] Masood R. . EphB4 provides survival advantage to squamous cell carcinoma of the head and neck. International journal of cancer. Journal international du cancer 119, 1236–1248, doi: 10.1002/ijc.21926 (2006). 16615113

[b4] Pasquale E. B. Eph receptor signalling casts a wide net on cell behaviour. Nat Rev Mol Cell Biol 6, 462–475, doi: 10.1038/nrm1662 (2005). 15928710

[b5] Noren N. K. & Pasquale E. B. Paradoxes of the EphB4 receptor in cancer. Cancer Res 67, 3994–3997, doi: 10.1158/0008–5472.CAN-07–0525 (2007). 17483308

[b6] Ferguson B. D. . The EphB4 receptor tyrosine kinase promotes lung cancer growth: a potential novel therapeutic target. PloS one 8, e67668, doi: 10.1371/journal.pone.0067668 (2013). 23844053 PMC3699624

[b7] Xia G. . EphB4 expression and biological significance in prostate cancer. Cancer Res 65, 4623–4632, doi: 10.1158/0008–5472.CAN-04–2667 (2005). 15930280

[b8] Kumar S. R. . Receptor tyrosine kinase EphB4 is a survival factor in breast cancer. Am J Pathol 169, 279–293, doi: 10.2353/ajpath.2006.050889 (2006). 16816380 PMC1698769

[b9] Xia G. . Up-regulation of EphB4 in mesothelioma and its biological significance. Clin Cancer Res 11, 4305–4315, doi: 10.1158/1078–0432.CCR-04–2109 (2005). 15958611

[b10] Hasina R. . Critical role for the receptor tyrosine kinase EPHB4 in esophageal cancers. Cancer research 73, 184–194, doi: 10.1158/0008–5472.CAN-12–0915 (2013). 23100466

[b11] Cromer A. . Identification of genes associated with tumorigenesis and metastatic potential of hypopharyngeal cancer by microarray analysis. Oncogene 23, 2484–2498, doi: 10.1038/sj.onc.1207345 (2004). 14676830

[b12] Lee Y. C. . Investigation of the expression of the EphB4 receptor tyrosine kinase in prostate carcinoma. BMC Cancer 5, 119, doi: 1471–2407–5–119 [pii] 10.1186/1471-2407-5-119 (2005). 16171530 PMC1266025

[b13] Sinha U. K. . The association between elevated EphB4 expression, smoking status, and advanced-stage disease in patients with head and neck squamous cell carcinoma. Arch Otolaryngol Head Neck Surg 132, 1053–1059, doi: 10.1001/archotol.132.10.1053 (2006). 17043250

[b14] Winter S. C. . Long term survival following the detection of circulating tumour cells in head and neck squamous cell carcinoma. BMC Cancer 9, 424, doi: 10.1186/1471-2407-9-424 (2009). 19961621 PMC3087340

[b15] Yavrouian E. J. . The significance of EphB4 and EphrinB2 expression and survival in head and neck squamous cell carcinoma. Arch Otolaryngol Head Neck Surg 134, 985–991, doi: 10.1001/archotol.134.9.985 (2008). 18794445

[b16] Bhatia S. . Knockdown of EphB1 receptor decreases medulloblastoma cell growth and migration and increases cellular radiosensitization. Oncotarget 6, 8929–8946, doi: 10.18632/oncotarget.3369 (2015). 25879388 PMC4496193

[b17] Nojiri K. . The proangiogenic factor ephrin-A1 is up-regulated in radioresistant murine tumor by irradiation. Exp Biol Med (Maywood) 234, 112–122, doi: 10.3181/0806-RM-189 (2009). 18997097

[b18] Kertesz N. . The soluble extracellular domain of EphB4 (sEphB4) antagonizes EphB4-EphrinB2 interaction, modulates angiogenesis, and inhibits tumor growth. Blood 107, 2330–2338, doi: 10.1182/blood-2005-04-1655 (2006). 16322467 PMC1895726

[b19] Ferguson B. D., Tretiakova M. S., Lingen M. W., Gill P. S. & Salgia R. Expression of the EPHB4 receptor tyrosine kinase in head and neck and renal malignancies–implications for solid tumors and potential for therapeutic inhibition. Growth Factors 32, 202–206, doi: 10.3109/08977194.2014.980904 (2014). 25391996 PMC4278660

[b20] Rangan S. R. A new human cell line (FaDu) from a hypopharyngeal carcinoma. Cancer 29, 117–121 (1972). 4332311 10.1002/1097-0142(197201)29:1<117::aid-cncr2820290119>3.0.co;2-r

[b21] Gioanni J. . Two new human tumor cell lines derived from squamous cell carcinomas of the tongue: establishment, characterization and response to cytotoxic treatment. Eur J Cancer Clin Oncol 24, 1445–1455 (1988). 3181269 10.1016/0277-5379(88)90335-5

[b22] Singleton K. R. . A receptor tyrosine kinase network composed of fibroblast growth factor receptors, epidermal growth factor receptor, v-erb-b2 erythroblastic leukemia viral oncogene homolog 2, and hepatocyte growth factor receptor drives growth and survival of head and neck squamous carcinoma cell lines. Mol Pharmacol 83, 882–893, doi: 10.1124/mol.112.084111 (2013). 23371912 PMC3608435

[b23] Pawlik T. M. & Keyomarsi K. Role of cell cycle in mediating sensitivity to radiotherapy. Int J Radiat Oncol Biol Phys 59, 928–942, doi: 10.1016/j.ijrobp.2004.03.005 (2004). 15234026

[b24] Brandsma I. & Gent D. C. Pathway choice in DNA double strand break repair: observations of a balancing act. Genome Integr 3, 9, doi: 10.1186/2041-9414-3-9 (2012). 23181949 PMC3557175

[b25] Ferguson B. D. . Novel EPHB4 Receptor Tyrosine Kinase Mutations and Kinomic Pathway Analysis in Lung Cancer. Sci Rep 5, 10641, doi: 10.1038/srep10641 (2015). 26073592 PMC4466581

[b26] Pradeep S. . Erythropoietin Stimulates Tumor Growth via EphB4. Cancer Cell 28, 610–622, doi: 10.1016/j.ccell.2015.09.008 (2015). 26481148 PMC4643364

[b27] Huang C. Y. . Sorafenib enhances radiation-induced apoptosis in hepatocellular carcinoma by inhibiting STAT3. Int J Radiat Oncol Biol Phys 86, 456–462, doi: 10.1016/j.ijrobp.2013.01.025 (2013). 23474115

[b28] Lee B. S. . Induced phenotype targeted therapy: radiation-induced apoptosis-targeted chemotherapy. J Natl Cancer Inst 107, doi: 10.1093/jnci/dju403 (2015). 25505252

[b29] Larsen A. B. . Activation of the EGFR gene target EphA2 inhibits epidermal growth factor-induced cancer cell motility. Molecular cancer research: MCR 5, 283–293, doi: 10.1158/1541-7786.MCR-06-0321 (2007). 17374733

[b30] Pasquale E. B. Eph receptors and ephrins in cancer: bidirectional signalling and beyond. Nature reviews. Cancer 10, 165–180, doi: 10.1038/nrc2806 (2010). 20179713 PMC2921274

[b31] Li X. . Up-regulation of EphA2 and down-regulation of EphrinA1 are associated with the aggressive phenotype and poor prognosis of malignant glioma. Tumour biology: the journal of the International Society for Oncodevelopmental Biology and Medicine 31, 477–488, doi: 10.1007/s13277-010-0060-6 (2010). 20571968

[b32] You J. . Effective photothermal chemotherapy using doxorubicin-loaded gold nanospheres that target EphB4 receptors in tumors. Cancer research 72, 4777–4786, doi: 10.1158/0008-5472.CAN-12-1003 (2012). 22865457 PMC3445780

[b33] Stahl S. . Inhibition of Ephrin B3-mediated survival signaling contributes to increased cell death response of non-small cell lung carcinoma cells after combined treatment with ionizing radiation and PKC 412. Cell death & disease 4, e454, doi: 10.1038/cddis.2012.188 (2013). 23303128 PMC3563978

[b34] Ang K. K. . Human papillomavirus and survival of patients with oropharyngeal cancer. N Engl J Med 363, 24–35, doi: 10.1056/NEJMoa0912217 (2010). 20530316 PMC2943767

[b35] *Transoral Surgery Followed By Low-Dose or Standard-Dose Radiation Therapy With or Without Chemotherapy in Treating Patients With HPV Positive Stage III-IVA Oropharyngeal Cancer*, (2013) (Date of access: 07/04/2016) https://clinicaltrials.gov/ct2/show/NCT01898494.

[b36] Garden A. S. . Radiation therapy (with or without neck surgery) for phenotypic human papillomavirus-associated oropharyngeal cancer. Cancer 122, 1702–1707, doi: 10.1002/cncr.29965 (2016). 27019396 PMC4873387

[b37] *Reduced-Dose Intensity-Modulated Radiation Therapy With or Without Cisplatin in Treating Patients With Advanced Oropharyngeal Cancer*, (2014) (Date of access: 16/04/2016) https://clinicaltrials.gov/ct2/show/NCT02254278.

[b38] Kelly J. R., Husain Z. A. & Burtness B. Treatment de-intensification strategies for head and neck cancer. Eur J Cancer 68, 125–133, doi: 10.1016/j.ejca.2016.09.006 (2016). 27755996 PMC5734050

[b39] Huang S. M., Bock J. M. & Harari P. M. Epidermal growth factor receptor blockade with C225 modulates proliferation, apoptosis, and radiosensitivity in squamous cell carcinomas of the head and neck. Cancer Res 59, 1935–1940 (1999). 10213503

[b40] Noberini R. . PEGylation potentiates the effectiveness of an antagonistic peptide that targets the EphB4 receptor with nanomolar affinity. PLoS One 6, e28611, doi: 10.1371/journal.pone.0028611 (2011). 22194865 PMC3237458

[b41] Al-Ejeh F. . Eph family co-expression patterns define unique clusters predictive of cancer phenotype. Growth Factors 32, 254–264, doi: 10.3109/08977194.2014.984807 (2014). 25410964

[b42] Kryeziu K. . Synergistic anticancer activity of arsenic trioxide with erlotinib is based on inhibition of EGFR-mediated DNA double-strand break repair. Molecular cancer therapeutics 12, 1073–1084, doi: 10.1158/1535-7163.MCT-13-0065 (2013). 23548265

[b43] Raju U. . Dasatinib, a multi-kinase inhibitor increased radiation sensitivity by interfering with nuclear localization of epidermal growth factor receptor and by blocking DNA repair pathways. Radiother Oncol 105, 241–249, doi: 10.1016/j.radonc.2012.08.010 (2012). 23010482

[b44] Banath J. P. & Olive P. L. Expression of phosphorylated histone H2AX as a surrogate of cell killing by drugs that create DNA double-strand breaks. Cancer Res 63, 4347–4350 (2003). 12907603

[b45] Muslimovic A., Nystrom S., Gao Y. & Hammarsten O. Numerical analysis of etoposide induced DNA breaks. PLoS One 4, e5859, doi: 10.1371/journal.pone.0005859 (2009). 19516899 PMC2689654

[b46] Zandi R., Larsen A. B., Andersen P., Stockhausen M. T. & Poulsen H. S. Mechanisms for oncogenic activation of the epidermal growth factor receptor. Cellular signalling 19, 2013–2023, doi: 10.1016/j.cellsig.2007.06.023 (2007). 17681753

[b47] Yu H. & Jove R. The STATs of cancer–new molecular targets come of age. Nat Rev Cancer 4, 97–105, doi: 10.1038/nrc1275 (2004). 14964307

[b48] Grandis J. R. . Constitutive activation of Stat3 signaling abrogates apoptosis in squamous cell carcinogenesis *in vivo*. Proc Natl Acad Sci USA 97, 4227–4232 (2000). 10760290 10.1073/pnas.97.8.4227 PMC18206

[b49] Jung M. . Human fibroblasts for large-scale “omics” investigations of ATM gene function. Advances in experimental medicine and biology 720, 181–190, doi: 10.1007/978-1-4614-0254-1_15 (2011). 21901628 PMC3744187

[b50] Cheema A. K. . Integrated analysis of ATM mediated gene and protein expression impacting cellular metabolism. Journal of proteome research 10, 2651–2657, doi: 10.1021/pr101243j (2011). 21322649

