# Supplementary material for: Enhancing radiosensitization in EphB4 receptor-expressing Head and Neck Squamous Cell Carcinomas
Source: Sci Rep. 2016 Dec 12;6:38792. doi: 10.1038/srep38792 (PMC5150255; doi:10.1038/srep38792)

**Title: Enhancing radiosensitization in EphB4 receptor-expressing Head and Neck Squamous Cell Carcinomas**

**Authors:** Shilpa Bhatia^1^, Kellen Hirsch^1^, Jaspreet Sharma^1^, Ayman Oweida^1^, Anastacia Griego^1^, Stephen Keysar^3^, Antonio Jimeno^3^, David Raben^1^, Valery Krasnoperov^4^, Parkash S. Gill^5^, Elena B. Pasquale^6^, Xiao-Jing Wang^7^, and Sana D. Karam^1*^

**Supplementary data**

**Supplementary table 1. Characteristics of HNSCC patient cases**

**Supplementary figure 1. EphB4 knockdown combined with ionizing radiation results in increased accumulation of HNSCC cells in the G2 phase of cell cycle.** Flow cytometry analysis show enhanced G2 cell cycle arrest in MSK-921 cells **(A, B)** and Fadu cells **(C, D)** following combined EphB4 knockdown and ionizing radiation. The cells were analyzed at 48-72 h post-radiation. Each experiment was repeated atleast two to three times. Representative plot is shown for each individual cell line.

**Supplementary figure 2. Combined EphB4 knockdown and ionizing radiation treatment enhances the percentage of p-H2AX positive HNSCC cells.** Flow cytometry analysis at 4-6 h post-radiation shows enhanced percentage of p-H2AX expressing Cal27 cells **(A)** and MSK-921 cells **(B)** after EphB4 downregulation and ionizing radiation compared to single agent treatment. Data represent mean±standard error from two independent experiments. *p<0.05

**Supplementary figure 3. EphB4 receptor knockdown decreases the levels of both phosphorylated and total EGFR.** Western blot analysis show a reduction in the levels of both phosphorylated EGFR and total EGFR following combined EphB4 downregulation and ionizing radiation exposure compared to each treatment alone in MSK-921 cells.

**Supplementary figure 4. The EphB4 receptor is expressed in HNSCC PDX tumors.** Western blot analysis shows that EphB4 protein is expressed at high to moderate levels in HNSCC PDX tumors.

**Supplementary figure 5. Targeting of EphB4 receptor shows no radiosensitization in CUHN022 HNSCC PDX model following high dose of fractionated radiation. (A, B)** Growth analysis of CUHN022 PDX tumors shows no substantial difference in tumor volume over time in mice administered with sEphB4-HSA+XRT compared to PBS+XRT. sEphB4-HSA was administered three times a week during the course of experiment. The symbol “↓” represent days when tumors were exposed to ionizing radiation (5 Gy/fraction). Data represent mean±standard deviation. Difference between the sEphB4-HSA+XRT versus PBS+XRT is not significant (NS), *p<0.05.

**Supplementary figure 6. sEphB4-HSA treatment decreases EphB4 tyrosine phosphorylation (activation) in HNSCC PDX tumors.** EphB4 phosphorylation is decreased significantly in CUHN004 tumors treated with sEphB4-HSA compared to PBS treatment as determined by ELISA assay. Data represent mean±standard deviation. ** p<0.001.

**Supplementary table 1**: Characteristics of HNSCC patient cases (Data Source: Keysar *et al*., 2013; RT: radiation therapy; Doc: Docetaxel; Cet: Cetuximab)

| **Cell line** | **Gender** | **Site** | **Initial stage** | **Type** | **Smoking** | **HPV status** | **Treatment modality** | **Disease status** |
| --- | --- | --- | --- | --- | --- | --- | --- | --- |
| CUHN013 | Female | Floor of mouth | T3N2cM0 | Primary | Yes | - | Surgery followed by carbotaxol+RT | Death due to rapid disease progression |
| CUHN004 | Male | Floor of mouth | T3N1M0 | Relapse | Yes | - | RT plus Doc and Cet | Tumor relapsed; patient is deceased |
| CUHN022 | Male | Tonsil | T2N2bM0 | Primary | No | + | Induction chemo with TPFx2 followed by RT+Cet | No disease evidence; patient is alive |


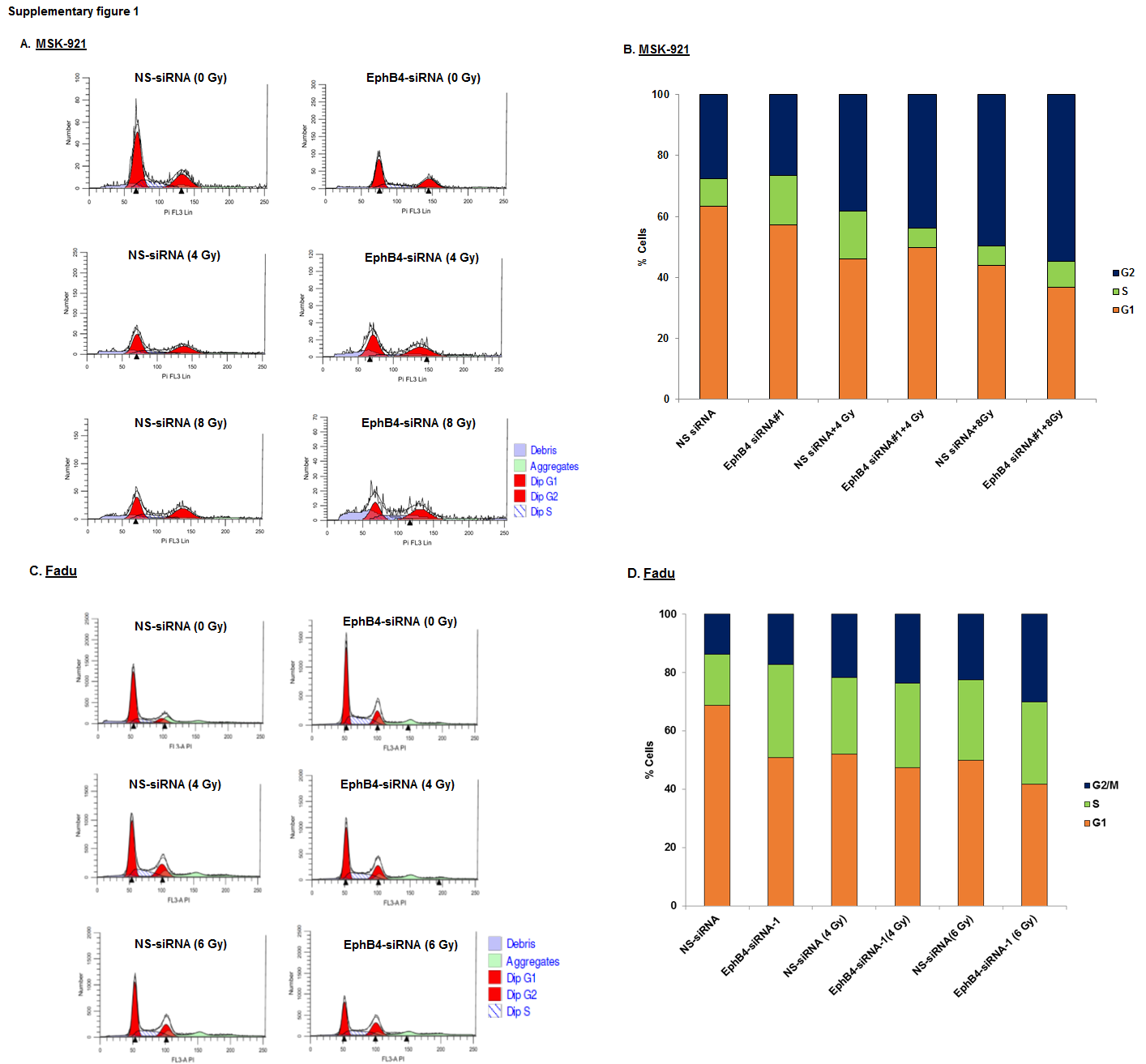

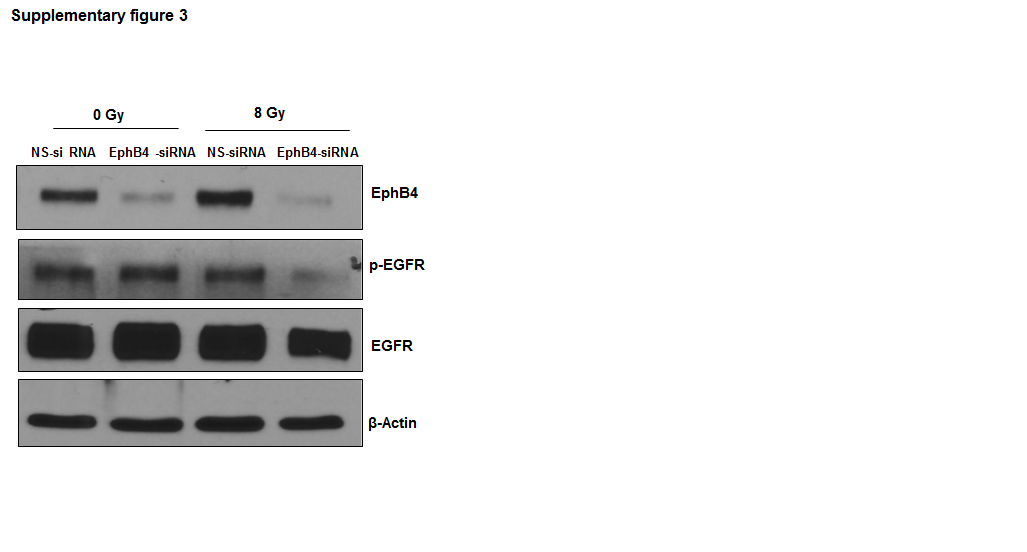

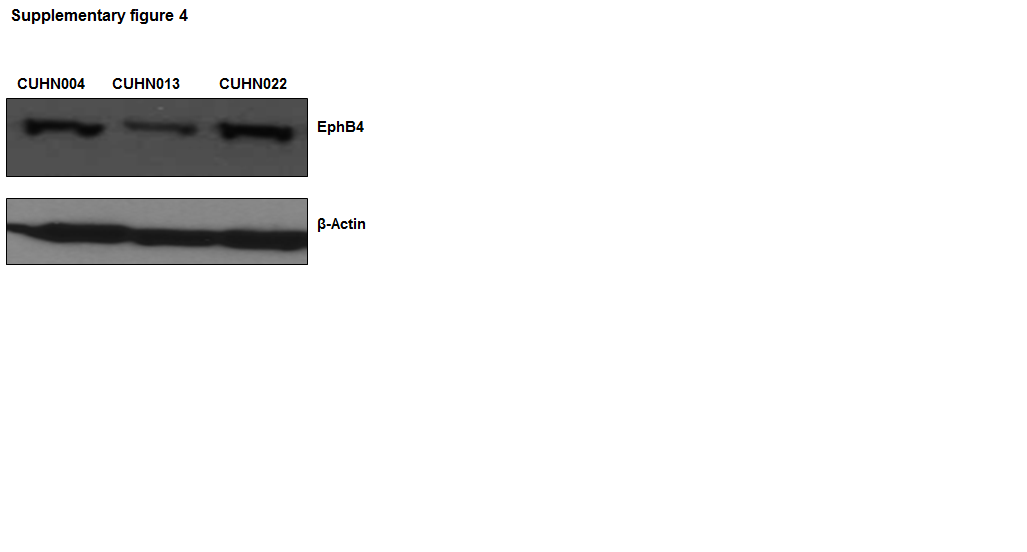

Supplement: Supplementary Data [file srep38792-s1.docx]
